# Dissecting the Extracellular Complexity of Neuromuscular Junction Organizers

**DOI:** 10.3389/fmolb.2019.00156

**Published:** 2020-01-10

**Authors:** Salvatore R. Guarino, Anselmo Canciani, Federico Forneris

**Affiliations:** The Armenise-Harvard Laboratory of Structural Biology, Department Biology and Biotechnology, University of Pavia, Pavia, Italy

**Keywords:** neuromuscular junctions, agrin, MuSK, LRP4, neurotrypsin, acetylcholine receptor, tyrosine kinase signaling

## Abstract

Synapse formation is a very elaborate process dependent upon accurate coordination of pre and post-synaptic specialization, requiring multiple steps and a variety of receptors and signaling molecules. Due to its relative structural simplicity and the ease in manipulation and observation, the neuromuscular synapse or neuromuscular junction (NMJ)—the connection between motor neurons and skeletal muscle—represents the archetype junction system for studying synapse formation and conservation. This junction is essential for survival, as it controls our ability to move and breath. NMJ formation requires coordinated interactions between motor neurons and muscle fibers, which ultimately result in the formation of a highly specialized post-synaptic architecture and a highly differentiated nerve terminal. Furthermore, to ensure a fast and reliable synaptic transmission following neurotransmitter release, ligand-gated channels (acetylcholine receptors, AChRs) are clustered on the post-synaptic muscle cell at high concentrations in sites opposite the presynaptic active zone, supporting a direct role for nerves in the organization of the post-synaptic membrane architecture. This organized clustering process, essential for NMJ formation and for life, relies on key signaling molecules and receptors and is regulated by soluble extracellular molecules localized within the synaptic cleft. Notably, several mutations as well as auto-antibodies against components of these signaling complexes have been related to neuromuscular disorders. The recent years have witnessed strong progress in the understanding of molecular identities, architectures, and functions of NMJ macromolecules. Among these, prominent roles have been proposed for neural variants of the proteoglycan agrin, its receptor at NMJs composed of the lipoprotein receptor-related protein 4 (LRP4) and the muscle-specific kinase (MuSK), as well as the regulatory soluble synapse-specific protease Neurotrypsin. In this review we summarize the current state of the art regarding molecular structures and (agrin-dependent) canonical, as well as (agrin-independent) non-canonical, MuSK signaling mechanisms that underscore the formation of neuromuscular junctions, with the aim of providing a broad perspective to further stimulate molecular, cellular and tissue biology investigations on this fundamental intercellular contact.

## Neuromuscular Junctions: Organization and Function

**Figure 1:**
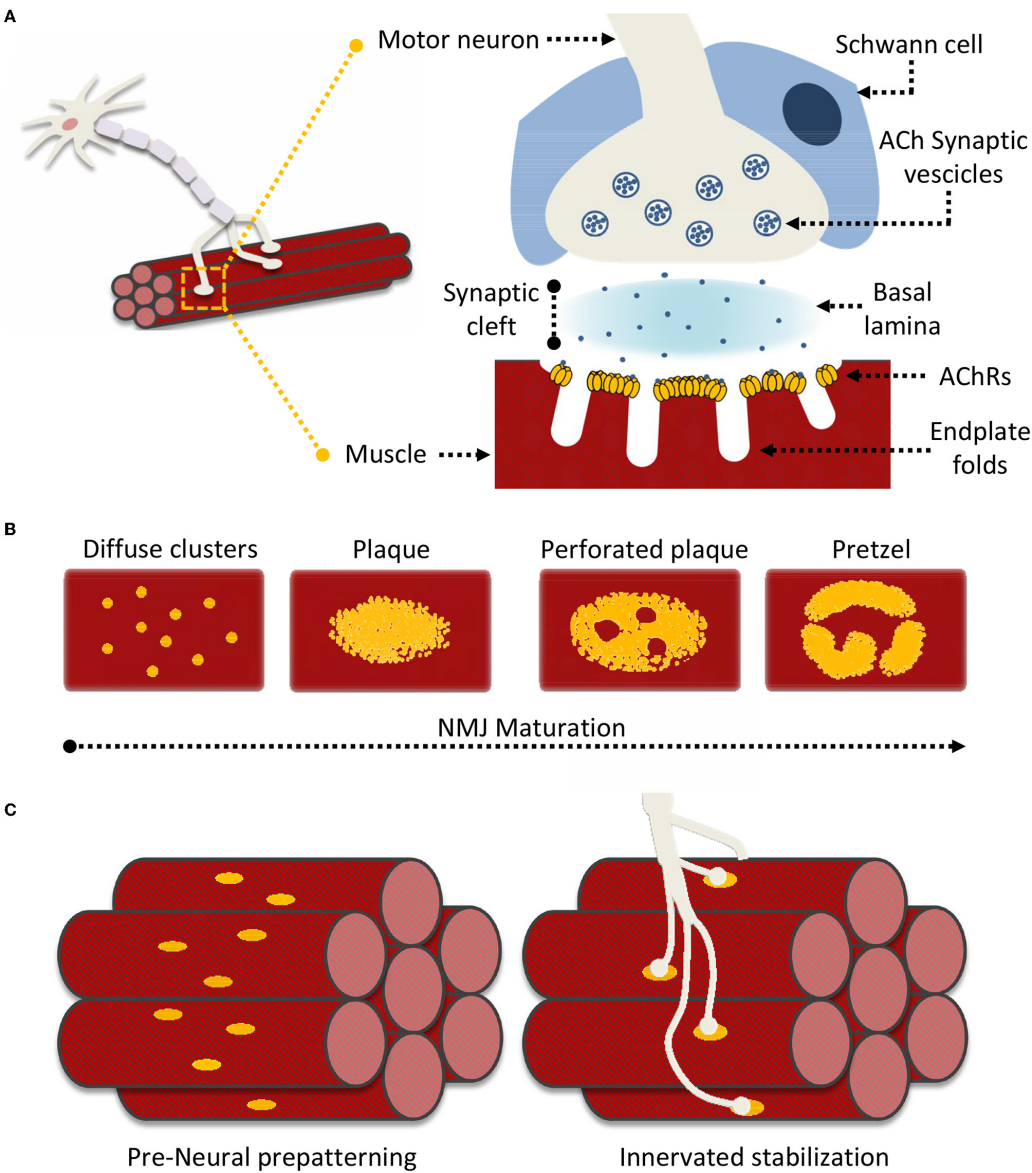
Neuromuscular Junction (NMJ) superstructure and maturation. **(A)** Schematic representation of the overall nerve-muscle interface; enlargement of the NMJ superstructure showing clustered acetylcholine receptors (AChRs) juxtaposed to the motor neuron terminal “embraced” by a supporting terminal Schwann cell. **(B)** General scheme of “plaque-to-pretzel” maturation; diffuse AChR clusters coalesce into plaques which then “perforate” and re-structure to give rise to the typical NMJ pretzel-like architecture. **(C)** During prepatterning “immature” AchR plaques align perpendicular to muscle fiber length. These guide outreaching neuronal branches and restrict/restrain contact formation. Motor neurons stabilize pre-existing contacted AChR hot-spots, non-innervated clusters are dispersed.

Neuromuscular junctions (NMJ) are essential contact points between the nervous system and muscular apparatus. They are characterized by specialized and distinguishing synapse architectures that juxtapose motor neuron presysnaptic terminals with post-synaptic muscle fibers (Figure 1A). This interface enables the reliable and efficient conversion of electrical impulses into chemical (neurotransmitter) messages, which in turn generate mechanical responses (Slater, 2017). In vertebrates, post-synaptic NMJ structures (endplates) present distinct membrane folds, extending into the muscle cell cytoplasm and resulting in increased post-synaptic membrane area, which improves the efficiency of signal transmission. The synaptic cleft encompassed between the nerve terminal and the muscle fiber spans around 50–100 nm and is filled by characteristic extracellular matrix (ECM) components that constitute the basal lamina, essential for NMJ formation, maturation and function (Wu et al., 2010; Slater, 2017). Schwann cells surround the synaptic cleft contributing to both NMJ maintenance, and neuronal guidance in post-injury re-innervation processes (Wu et al., 2010; Rogers and Nishimune, 2017; Slater, 2017).

Signaling at the NMJ depends on the neurotransmitter acetylcholine (ACh), which enables the conversion of neuronal electrical signals into muscle-derived mechanical responses via downstream calcium-induced calcium release (Li L. et al., 2018). Unsurprisingly, the muscle fiber response to ACh is proportional to the number of nicotinic acetylcholine receptors (AChRs) located in close proximity to the post-synaptic terminal. Indeed AChR density at the NMJ reaches 10,000 AChRs/μm^2^, while drastically dropping to <10 AChRs/μm^2^ in the extra-junctional muscle membrane (Hartzell and Fambrough, 1972; Heuser and Salpeter, 1979). The vast majority of junctional AChRs are located at the mouth of the endplate folds, closest to the point of ACh release (Fertuck and Salpeter, 1974). The pit of these same folds is lined with densely-packed voltage-gated sodium channels (Na_V_1s), responsible for propagating the AChR-triggered depolarization across the muscle fiber. Thus, the NMJ membrane folds act as “signal amplifiers” for ACh and highlight how the structural organization of the NMJ is as important as its constituents for adequate signal transmission (Haimovich et al., 1987; Slater, 2017; Li L. et al., 2018).

## NMJ Formation: From AChR Clusters to Functional Interface

The formation and organization of NMJs is an elaborate process analogous to that responsible for central nervous system (CNS) synapses. Anterograde (presynaptic) and retrograde (post-synaptic) signals shepherd neuron-muscle interplay through distinct developmental steps. This process is characterized, throughout, by post-synaptic modifications in AChR synthesis, distribution, density, and a distinct modification of the muscle fiber membrane, at sites of neuronal interface (Kummer et al., 2006; Slater, 2017; Li L. et al., 2018). During embryonic development, immature muscle cells (myoblasts) fuse to form muscles where AChRs are initially diffuse throughout the muscle membrane (Sanes and Lichtman, 2001; Figure 1B). However, as embryonic development progresses, isolated AChR clusters start to coalesce into distinct oval-like plaques that accumulate in a tight band at the center of the muscle fiber in a process known as pre-patterning (Kummer et al., 2006; Figure 1C). These plaques are the result of both pre- and post-synaptic differentiation processes, as their formation can be induced neuronally at contact sites, or occur “spontaneously” as aneural AChR “hotspots” on the muscle fiber (Fischbach and Cohen, 1973; Anderson and Cohen, 1977).

While initial neural vs. aneural temporal contribution to pre-patterning is unclear, the predominant theory holds that aneurally-formed plaques might serve as guidance signals for outreaching motoneurons. Regardless, the process ultimately yields a restricted (characteristic) distribution of nerve-muscle contacts along an axis perpendicular to fiber length. Formation of these primitive contacts initiates processes of plaque dispersal, eliminating AChR clusters lacking a neural interface while increasing receptor density at contact sites (Sanes and Lichtman, 2001). Subsequently, as NMJs mature, the oval-like plaque organization is lost in favor of the more pretzel-like appearance characteristic of fully formed NMJs (Figure 1B). This process is accompanied by a host of structural modifications as the post-synaptic muscle membrane reconfigures itself to form the NMJ endplate folds (Marques et al., 2000; Sanes and Lichtman, 2001). Signaling cues directing NMJ maturation originate from multiple sources. These include both the pre- and post-synaptic terminals, the surrounding Schwann cells, and even basal lamina components such as laminins (Rogers and Nishimune, 2017; Li L. et al., 2018). AChR distribution and synthesis varies throughout this process in response to this elaborate network of signaling cues. In this context, one of the better described processes underlying AChR clustering, essential to NMJ formation and maintenance, is the “canonical” agrin-dependent pathway. This involves signaling through the two muscle bound transmembrane co-receptors LRP4 (low-density lipoprotein receptor 4) and MuSK (muscle specific kinase), and possibly proteolytic regulation of agrin via the nervous system serine protease Neurotrypsin (NT) (Butikofer et al., 2011; Zong et al., 2012; Burden et al., 2018; Li J. et al., 2018; Li L. et al., 2018).

## The Molecular Architecture of Agrin, a Key Signaling Molecule for NMJ Formation and Stability

Agrin is a very large, extensively glycosylated proteoglycan spanning more than 2'000 aminoacids with an estimated molecular weight of 500 kDa. While it is best known for its essential function in NMJ formation, agrin is expressed in a variety of different tissues where it plays roles often associated with cell motility, specialization, and proliferation (Daniels, 2012; Mccarthy, 2015). Thus, agrin is found contributing to processes as diverse as CNS plasticity (Molinari et al., 2002; Matsumoto-Miyai et al., 2009; Mccroskery et al., 2009; Porten et al., 2010; Daniels, 2012), regulation of the hematopoietic stem cell niche (Mazzon et al., 2011, 2012), lymphocyte mediated immune cell response (Khan et al., 2001; Zhang et al., 2006; Jury and Kabouridis, 2010; Kabouridis et al., 2012), cell adhesion (Steiner et al., 2014; Chakraborty et al., 2015; Anselmo et al., 2016), and even modulation of cartilage and bone formation (Hausser et al., 2007; Eldridge et al., 2016).

**Figure 2:**
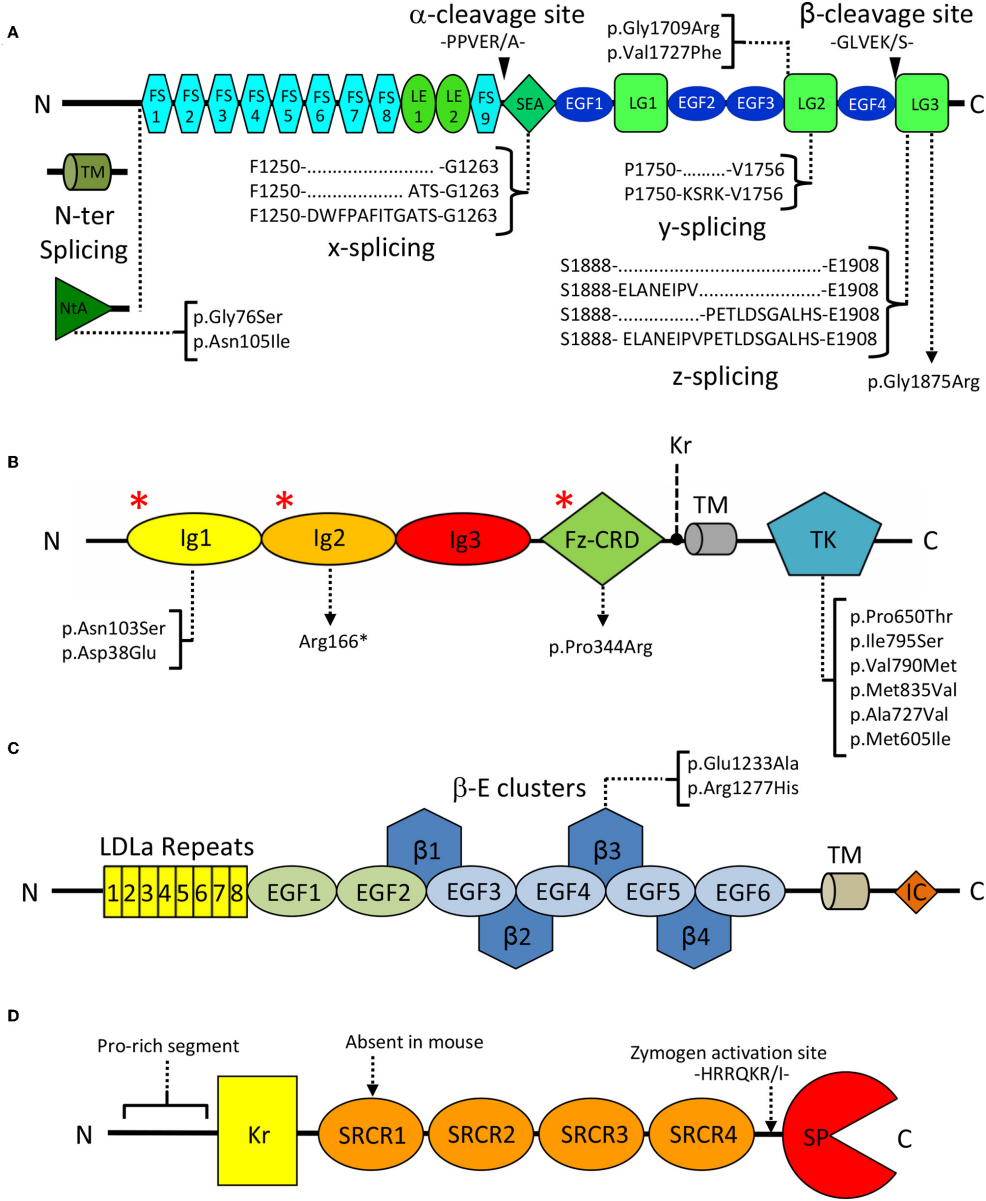
Domain organization of key extracellular NMJ organizers. **(A)** Agrin: FS, Follistatin-like; LE, Laminin EGF-like; SEA, sperm, enterokinase and agrin; EGF, Epidermal growth factor like; LG, Laminin globular. Curly brackets show splicing sites and variants. Black arrowheads indicate Neurotrypsin cleavage sites α and β. Square brackets map missense pathogenic CMS mutations to the affected domains. **(B)** MuSK: Ig, Immunoglobulin-like; Fz-CRD, Frizzled-like cysteine-rich; TK, Tyrosine Kinase; Kr, Kringle; TM, transmembrane region. Square brackets map missense pathogenic CMS mutations to the affected domains, and red asterisks (*) indicate the epitopes recognized by autoantibodies in autoimmune MG. **(C)** LRP4: LDLa, Low density lipoprotein class A; EGF, Epidermal growth factor like; β: β-propeller; TM: transmembrane, IC: Intracellular. Square brackets map missense pathogenic CMS mutations. **(D)** Neurotrypsin: Kr, Kringle; SRCR, Scavenger receptor cysteine rich; SP, Serine-protease. The zymogen-activating region is indicated by a dashed arrow.

In spite of its multiple roles, agrin's domain composition is highly conserved, and includes 9 follistatin-like (FS), 2 laminin EGF-like (LE), 4 EGF-like (EGF), 1 SEA (sperm protein, enterokinase, and agrin), and 3 laminin globular (LG1-LG3) domains (Figure 2A). Interestingly this last C-terminal LG1-LG3 region was reported sufficient for inducing AChR clustering, mimicking the effect of the full length proteoglycan (Cornish et al., 1999). Three key splice sites (N-ter, y, and z) are present on agrin and collectively result in the expression of different isoforms that display a certain degree of tissue specificity and functional difference (Hoch et al., 1993; Bezakova and Ruegg, 2003). N-terminal splicing differentiates agrin's transmembrane (TM) and secreted variants, respectively predominant outside and inside the CNS. The former bears a transmembrane region and a small intracellular domain of unknown function, while the latter carries the NtA domain known to mediate anchoring to ECM laminins at the NMJ (Denzer et al., 1997; Bezakova and Ruegg, 2003). Splicing at the y site does not result in altered domain composition but corresponds to a 4-aminoacid insertion, at the LG2 domain (Figure 2A), known to modulate binding to heparin and to α-dystroglycan (Bezakova and Ruegg, 2003). Similarly, splicing at the z site on the LG3 domain results in the addition of 8, 11, or 19 residues. Interestingly, the z8 and z11 variants do not have overlapping sequences, and the z19 variant results from the cumulative insertion of the z8 and z11 sequences (Figure 2A). Both the z8 and z19 inserts are of particular importance as they contain 8 aminoacids pivotal to agrin's AChR clustering capabilities (Bezakova and Ruegg, 2003; Zong et al., 2012). Notably, agrin C-terminal constructs deprived of the z8 insertion are incapable of inducing MuSK signaling and AChR clustering. Conversely, short fragments encompassing the LG3 domain alone carrying the z8 insertion are sufficient to induce AChR clustering, albeit with very low efficiency (Mcmahan et al., 1992; Ferns et al., 1993; Gesemann et al., 1995). Agrin isoforms containing any of these three insertions are predominantly expressed in neuronal cells and are virtually absent in non-neuronal tissues (Hoch et al., 1993; Bezakova and Ruegg, 2003). An ulterior splice site (x), corresponding to a 3- or 12- aminoacid insertion at the C-terminal end of the SEA domain (Figure 2A), has been documented for rat agrin. However, its functional significance and conservation across species have yet to be assessed (Hoch et al., 1993; Ohno et al., 2017).

Regardless of alternative splicing, agrin remains a heavily modified protein with several predicted N-glycosylation sites, two serine/treonine-rich regions likely to accommodate O-linked glycosylations, and a known O-fucosylation on Ser1835 (Ser1726 on rat agrin) in the EGF4 domain (Rupp et al., 1991; Tsim et al., 1992; Groffen et al., 1998; Kim M. L. et al., 2008). The extensive glycosylation of agrin, however, depends predominantly on the attachment of heparan sulfate (HS) and chondroitin sulfate (CS) glycosaminoglycan side-chains to its N-terminal half. These account for the impressive MW of full length agrin, roughly doubling what can be estimated from the aminoacid sequence alone (~220 kDa) (Halfter, 1993; Burg et al., 1995; Winzen et al., 2003). Interestingly, these HS/CS modifications are likely to play a functional role in agrin-mediated signaling as they were seen to modulate agrin proteolysis by the nervous system serine-protease Neurotrypsin (NT) (Reif et al., 2007; Gisler et al., 2013).

## A Structural Glimpse of Agrin

Spanning more than three decades, literature on agrin extensively addresses its biological relevance and its AChR clustering properties. Comparatively, the pertinent structural information is significantly scarcer, likely as a consequence of agrin's size and extensive post-translational modifications. Nonetheless what has been described provides a glimpse of agrin's overall structure, and yields invaluable insights into its structure-function relationship.

**Figure 3:**
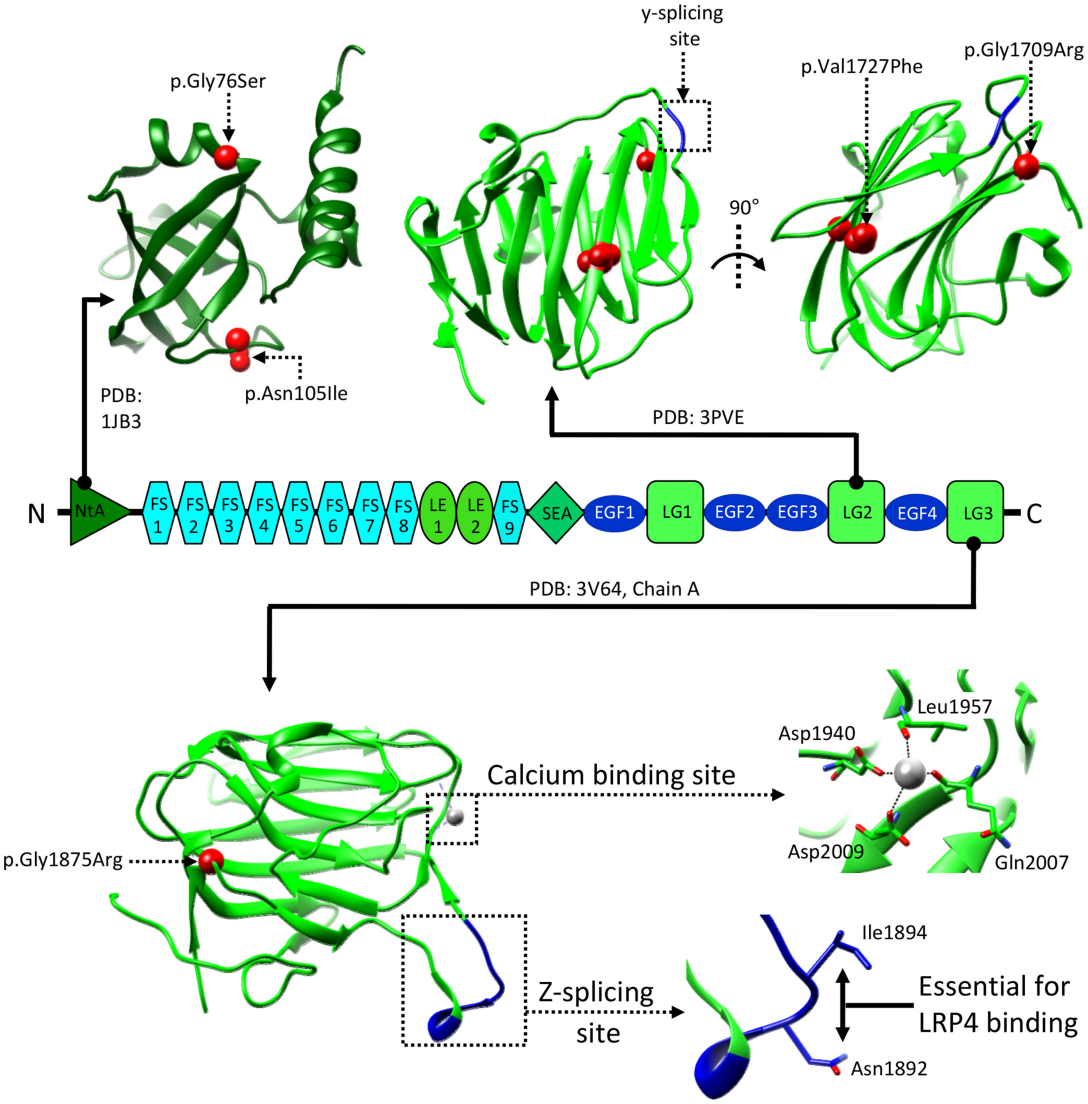
Structural information about agrin. The structure of the NtA, LG2, and LG3 domains of agrin are shown in correspondence to the overall domain organization. Reported human pathogenic mutations are indicated and mapped on available reference structures as red spheres. Splicing insertion sites y and z are boxed and shown in blue. Salient features of the LG3 domain are enlarged to show the residues mediating z8 binding to LRP4, and the Ca^2+^ binding site. Residue numbering refers to the human protein (uniprot: O00468), corresponding residues on the reference structures are as follows: LG2 residues Val1727 and Gly1709, correspond to Val1548 and Gly1530, respectively. LG3 residues Gly1875, Asn1892, Ile1894, Asp1940, Leu1957, Gln2007, and Asp2009, correspond to Gly1766, Asn1783, Ile1785, Asp1820, Leu1837, Gln1887, and Asp1889, respectively.

Initial low resolution investigations of agrin's structure revealed the globular nature of both the N-terminal NtA domain and LG1-3 C-terminal domains, and highlighted the presence of several HS/CS chains. Surprisingly, the region connecting the NtA to the LG cluster was seen to be less clearly structured, appearing as a long and flexible rope/rod-like segment (Denzer et al., 1998). Accordingly, high resolution structural information on agrin is mostly confined to the globular regions, as only the laminin-binding NtA domain (Stetefeld et al., 2001; Mascarenhas et al., 2005; Mcfarlane and Stetefeld, 2009) and the last two LG domains [LG2 (doi: 10.2210/pdb3PVE/pdb) and LG3 (Stetefeld et al., 2004; Zong et al., 2012), respectively] have been crystallized (Figure 3). Of these, the structures of the LG3 domain are more informative on agrin's structure-function relationship with its NMJ receptor LRP4:MuSK. Notably, the z-spicing insertion site was seen to be a hyperflexible region stabilized only when the z8 variant was crystallized in complex with the first extracellular β-propeller domain (β-E1) of LRP4 (Stetefeld et al., 2004; Zong et al., 2012). Interestingly, the agrin:LRP4 interaction was seen to be heavily reliant on an NxI motif present only on z8/z19 inserts, explaining their unique proficiency in initiating AChR clustering (Ferns et al., 1992; Hoch et al., 1993; Zong et al., 2012).

The agrin LG3 domain structures also offer insights into Ca^2+^ dependency, reported for agrin-mediated AChR clustering (Bloch, 1983; Wallace, 1988). Notably, an essential Ca^2+^ binding site was discovered in proximity to the z-insert loop (Tseng et al., 2003; Stetefeld et al., 2004). Interestingly, interaction of the agrin LG3 with Ca^2+^ was seen to “rigidify” the domain, likely restraining the mobility of the hyperflexible z-insert loop (Figure 3). An alteration which could, in turn, facilitate binding to LRP4 and result in increased AChR clustering (Tseng et al., 2003; Stetefeld et al., 2004; Zong et al., 2012). However, the Ca^2+^ affinity estimated for this domain is low (~10 mM) (Tseng et al., 2003), and significantly inferior (~30-fold) to the half-maximal concentration (~0.2–0.3 mM) reported to affect AChR clustering in cell-based experiments (Bloch, 1983; Wallace, 1988). This suggests a greater cooperativity centered on Ca^2+^ than can be explained by the binding to the agrin LG3 domain alone. While it is reasonable to assume significant contributions from the remaining agrin domains, this cooperative effect likely extends beyond a single NMJ component. Indeed, the structure of the agrin LG3-z8:LRP4-β-E1 complex shows how Ca^2+^ ions bind to both agrin and LRP4 domains likely contributing to the stabilization and dimerization of the complex (Zong et al., 2012).

## MuSK: One of the Keystones of AChR Clustering and NMJ Development

As mentioned previously, AChR clustering occurs during two distinct phases of NMJ development, respectively in a neurally dependent or independent fashion. Of these the former relies on the better understood “canonical” activation of MuSK by LRP4:agrin. MuSK was originally discovered in the electric organ of *Torpedo californica*, in which the post-synaptic terminal presents an abnormal abundance of proteins involved in muscle differentiation (Jennings et al., 1993). While also expressed in the central nervous system (Garcia-Osta et al., 2006) and in sperm cells (Kumar et al., 2006), this protein is best known for its critical functional role in skeletal muscles. There, its activation triggers a complex pathway culminating with the formation of NMJs dependent upon the correct juxtaposition of AChR clusters, on the muscle cell membrane, with motor neuron nerve terminals (Burden et al., 2018). The importance of MuSK in this process is evident, as its knock-out in mice results in respiratory failure and neonatal lethality caused by a lack of functional NMJs (Dechiara et al., 1996).

MuSK is a single-pass type I transmembrane receptor tyrosine kinase of 140 kDa localized on the muscle membrane. It displays a structural organization typical of receptors of the protein tyrosine kinase (PTK) superfamily. As such, it is composed of a multi-domain N-terminal extracellular region (ectodomain), a transmembrane helix, and a cytosolic segment. This latter element incorporates a unique juxtamembrane region, as well as MuSK intracellular tyrosine kinase (TK) catalytic domain (Figure 2B).

The ectodomain of human MuSK is characterized by a distinguishing domain organization typical of scavenger receptors. It is composed of three immunoglobulin-like domains (Ig1-Ig3) followed by a Frizzled-like cysteine-rich domain (Fz-CRD). In fish, amphibians and birds an additional kringle domain is found in between the Fz-CRD and the transmembrane helix (Burden et al., 2018; Figure 2B).

## “Canonical” NMJ Signaling Requires MuSK Activation

**Figure 4:**
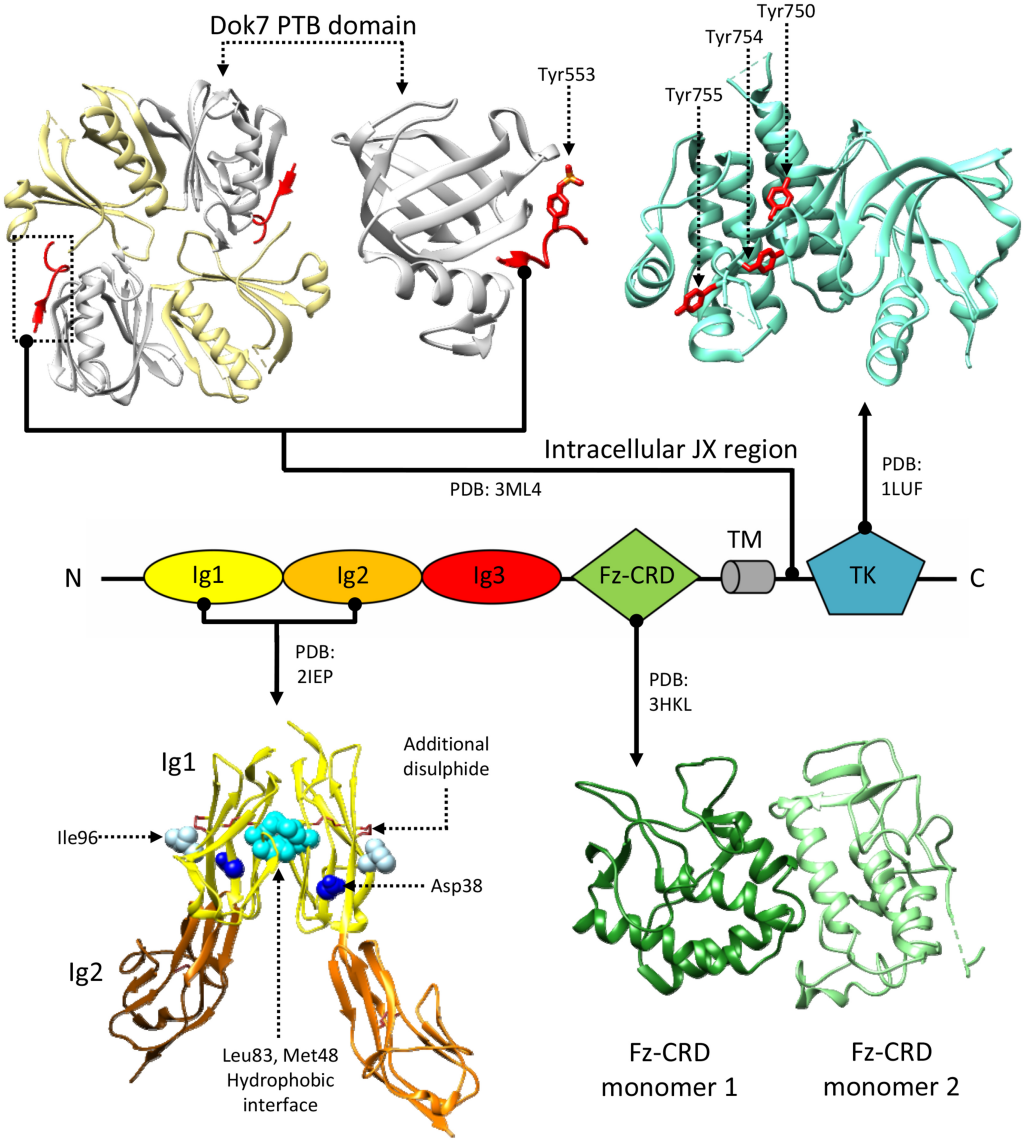
Structural information about MuSK. MuSK interacts with the Dok7 (top-left) phosphotyrosine-binding domain (PTB, white) via a phosphorylated tyrosine (Tyr553) on its intracellular juxtamembrane (JX) region (red). The tyrosine kinase (TK) domain (top right) mediates intracellular signaling after phosphorylation of Tyr750, Tyr754, and Tyr755 (shown in red), located on a regulatory loop. Extracellular interactions with LRP4 are mediated by MuSK Ig domains. A crystal structure of an Ig1-Ig2 MuSK dimer (bottom left) shows a hydrophobic dimerization interface (cyan) reliant upon aminoacids Met48 and Leu83. Residues Asp38 (blue) and Ile96 (white), on the exposed surfaces of the Ig1 domains, are respectively involved in congenital myasthenia and LRP4 binding. Disulphide bonds stabilize both the Ig1 and Ig2 domain structures, the former has an additional disulphide bridge. Unlike the Ig domains, the function of the Fz-CRD domain (bottom-right) is extensively debated. The crystal structure presents two alternative conformations packed in an asymmetric dimer. Residue numbering refers to the reference structures.

While the extracellular portion of MuSK is extensively involved in protein-protein interactions, the intracellular juxtamembrane region and TK domain are ultimately responsible for MuSK-mediated signaling. Notably, this juxtamembrane segment presents a phosphorylated tyrosine residue conserved in MuSK orthologs (Tyr553 in humans), that is recognized by the adapter protein Dok7 in a process crucial for MuSK activation (Herbst and Burden, 2000; Till et al., 2002; Figure 2B). It is unclear as to whether the initial phosphorylation of this tyrosine relies on independent MuSK basal activity, partial MuSK activation, or even a currently undetermined heterologous kinase. Regardless, binding of Dok7 to this residue results in Dok7 dimerization, facilitating the subsequent trans-phosphorylation of three other tyrosines (Tyr750, Tyr754, and Tyr755) located in the TK domain of MuSK (Till et al., 2002; Bergamin et al., 2010). In line with the characteristic RTK-family features of MuSK TK domain, these three residues are located in a TK activation loop (Figure 4; Till et al., 2002). Thus, when un-phosphorylated, these residues inhibit the TK active site, in a similar manner to the regulatory mechanism described for the insulin receptor TK domain (Hubbard et al., 1994). The autophosphorylation of MuSK TK domain, with concomitant Dok7 activation, is the trigger for initiating the downstream signaling cascade that ultimately induces AChR clustering (Hopf and Hoch, 1998). This process is mediated by a membrane-anchored intracellular scaffolding protein, with E3 ubiquitin ligase activity, named receptor-associated protein of the synapse (Rapsyn). This molecule plays an important role in establishing those networks that anchor AChRs to the muscle membrane and are ultimately responsible for the correct clustering of AChRs (Ramarao et al., 2001; Zuber and Unwin, 2013; Li et al., 2016; Burden et al., 2018). Interestingly, studies have shown that MuSK can interact with Rapsyn via an unidentified accessory protein, provisionally named receptor-associated transmembrane linker (RATL). It has been proposed that this MuSK:RATL:Rapsyn interaction could involve the extracellular region of MuSK, specifically the Fz-CRD and its adjacent juxtamembrane region (Zhou et al., 1999). While it is clear that Rapsyn is activated after MuSK dimerization, the underlying mechanisms remain poorly understood. A very recent report indicates that agrin:LRP4-mediated MuSK activation induces tyrosine phosphorylation of Rapsyn, which is required for its self-association and E3 ligase activity (Xing et al., 2019). This reveals an intriguing scenario where the initial trigger of the signaling cascade (MuSK) directly induces a multitude of cellular events, including the ultimate activation of the protein that clusters AChRs on the post-synaptic membrane.

## The Flexible MuSK Ectodomain Mediates Intricate, But Essential, Interactions

The general complexity of MuSK activation is reflected in the associated extracellular conformational rearrangements that involve and coordinate dimerization of its ectodomain, the co-receptor LRP4, and the agrin C-terminal region. Although a precise stoichiometry of agrin-free and agrin-bound LRP4:MuSK complexes has not been defined, evidence for low-level agrin-independent MuSK basal activity (Wu et al., 2010), suggests the presence of RTK homodimeric assemblies within the latent receptor:co-receptor complexes. Indeed, myotubes derived from MuSK knock-out mice fail to display both neural and aneural AChR clusters, fundamental during pre-neural phases (Zhou et al., 1999).

A crystal structure of MuSK Ig1-Ig2 domains (Stiegler et al., 2006), an extracellular fragment fundamental for RTK activation found sufficient to recover AChRs clustering (Zhou et al., 1999), supports a possible constitutive dimeric receptor assembly. In this structure, the two Ig1 domains interact, contacting one-another exclusively through hydrophobic residues (Figure 4). Besides showing the typical organization of intermediate set (I-set) of the immunoglobulin superfamily, with their canonical folds characterized by two anti-parallel β sheets connected by a disulphide bond, MuSK Ig1 domains also contain a second disulphide bond, highly conserved in MuSK orthologs and absent in other I-set Ig domains. This extra disulphide bond is a unique feature of MuSK Ig1, whose correct formation was found to be critical for proper folding of this domain (Stiegler et al., 2006) (Figure 4). Mutagenesis experiments revealed that alterations in the aminoacid network involved in the Ig1:Ig1 interface impairs MuSK phosphorylation (Stiegler et al., 2006), supporting the hypothesis of a stable MuSK homodimerization site at the Ig1 domain. However, solution studies using the same MuSK construct contradictorily showed the presence of monomers (Stiegler et al., 2006). Subsequent mutagenesis studies also highlighted the importance of the MuSK Ig1 in heteromeric contacts with LRP4. In particular, the presence of a surface exposed residue (Ile96, Figure 4) was found essential to LRP4:MuSK interactions (Zhang et al., 2011). Collectively, the available biochemical and structural data highlight multiple critical roles for the MuSK Ig1 domain. However, they do not provide a conclusive description of constitutive and activated MuSK conformations nor of any structural arrangements for its complex with LRP4.

Similarly, the role of the MuSK Fz-CRD is still unclear, as it appears to be dispensable for agrin-dependent NMJ signaling but not for the stabilization of newly-generated AChR clusters on muscle cell membranes. Indeed, full-length MuSK constructs lacking this domain fully retain their capability of inducing signaling and AChR clustering upon agrin stimulation, but show alterations in binding to Rapsyn (Zhou et al., 1999). Similar domains found in frizzled receptors constitute key elements of Wnt signaling pathways and play fundamental roles in ligand recognition (Bhanot et al., 1996; Janda et al., 2012). As such it could be possible to envisage a similar ligand-binding role for the MuSK Fz-CRD a hypothesis arguably investigated by several experiments addressing Wnt-mediated AChR clustering (Jing et al., 2009; Zhang et al., 2012a; Messeant et al., 2015, 2017; Remedio et al., 2016). It should be noted, however, that the Fz-CRD of MuSK shares less than 20% sequence identity with the other Frizzled CRDs.

Nonetheless, the structure of the MuSK Fz-CRD adopts an architecture characteristic of Frizzled CRDs. As such, it displays two N-terminal β-strands, an α-helical packing typical of this type of domains, and 5 disulphide bonds that stabilize the overall domain conformation (Figure 4; Stiegler et al., 2009). Interestingly, in the only reported MuSK Fz-CRD structure, two domains adopting different conformations are found asymmetrically arranged to form a homodimer. This could possibly suggest structural contributions to the conformational rearrangements enabling TK activation and/or downstream interactions with Rapsyn. The hypothesis that Fz-CRD dimers may have physiological roles is supported by the functional dimeric arrangements of distant homologous Frizzled-3 in *Xenopus laevis* (Carron et al., 2003). However, as was the case for the MuSK Ig1 domain, follow-up data highlighted only the presence of stable monomers in solution (Stiegler et al., 2009).

## LRP4, an Usually Complex Co-receptor Molecule

A distinguishing feature of MuSK activation lies in its inability to directly bind agrin in the extracellular space. Thus, an indispensable co-receptor (LRP4) is required to mediate agrin-induced RTK signaling (Kim N. et al., 2008; Zhang et al., 2008). Agrin binding to LRP4 occurs with picomolar affinity, and induces allosteric extracellular changes in the LRP4:MuSK receptor:co-receptor assembly (Hopf and Hoch, 1998; Zhang et al., 2011). Interestingly, MuSK and LRP4 form a significantly stable receptor:co-receptor complex, and can interact to form hetero-oligomeric assemblies also in the absence of agrin (Zhang et al., 2011).

LRP4 is a 220 kDa single-pass type I transmembrane protein belonging to the low-density lipoprotein-related receptor (LRP) family. Although LRPs are primarily known to perform metabolic functions such as lipid transport (Hussain, 2001), they also play important roles in signaling pathways (Herz, 2001; May et al., 2007). LRP4 is located on the muscle membrane and it constitutes an essential co-receptor component for agrin-dependent MuSK signaling during NMJ development. LRP4 has important implications in development as mice embryos lacking LRP4 exhibit important defects in limbs and organs including lungs and kidneys. As such, in a fashion analogous to their MuSK knock-out counterparts, LRP4 knock-out mice die at birth as a result of respiratory complications (Weatherbee et al., 2006).

Direct recognition of agrin by LRP4 is a crucial step in MuSK signaling, however the underlying mechanisms and structural rearrangements leading to MuSK activation remain unclear (Kim N. et al., 2008; Zhang et al., 2008). From a structural point of view, LRP4 displays a large extracellular region characterized by a multitude of folded domains, followed by a single transmembrane helix and a small cytoplasmic intracellular domain that was found dispensable for NMJ function (Gomez and Burden, 2011; Figure 2C). The LRP4 ectodomain is composed of eight LDLa (LDL class A) domain repeats, followed by two EGF-like domains and a cluster of four consecutive 6-bladed YWTD β-propeller-EGF domain repeats (β-E1-4). These precede a heavily glycosylated Ser/Thr-rich region in close proximity to the transmembrane helix. Folding of this complex ectodomain architecture requires multiple specialized chaperoning machineries. The 6-bladed YWTD β-E domain architecture constitutes an interdependent module often found in LRPs and also in other extracellular protein receptors (Springer, 1998; Takagi et al., 2003) and depends on the activity of the Mesoderm Development protein (MESD) (Chen et al., 2011; Collins and Hendrickson, 2011). Likewise, the N-terminal LDLa domain cluster requires dedicated assistance by the receptor-associated protein (RAP) (Jensen et al., 2009; Singhal and Martin, 2011). The precise molecular functions of those eight N-terminal LDLa domains are still matter of debate. They were reported to play roles in the differentiation of the presynaptic terminal through retrograde responses (Yumoto et al., 2012). However, to date, no presynaptic LDLa partner/receptor has been identified.

Possible insights into the functional roles of LRP4's LDLa repeats can be hypothesized based on what is known regarding the LDL receptor (LDLR). In LDLR, a similarly elongated set of seven LDLa repeats is known to constitute an essential “*molecular belt*” for the binding of apolipoprotein ligands (Russell et al., 1989; Rudenko et al., 2002). While the exact structural underpinnings guiding the LDL-apolipoprotein interaction remain unclear, it is known that the LDLa repeats exhibit negative charges (at neutral pH) that facilitate/mediate the interaction with the positive charges exposed on apolipoproteins (Innerarity et al., 1987; Brown et al., 1997). Then, upon ligand:receptor complex internalization, the endosomal pH triggers ligand release, inducing conformational changes that result in the LDLa repeats interacting with the β-propeller-EGF region (Rudenko et al., 2002). Therefore, one could imagine a similar mechanism for LRP4, with the LDLa repeats mediating internalization of ligands, such as Ca^2+^ (Brown et al., 1997) and agrin (Zhang et al., 2011), which could modulate intracellular signaling cascades (Tseng et al., 2003). Interestingly, biochemical studies have shown that only a subset of LRP4s LDLa repeats (6–8) is required to stabilize binding with agrin (Zhang et al., 2011). Such an observation raises questions regarding the actual function of such a long LDLa repeat cluster at the N-terminus of this co-receptor molecule.

**Figure 5:**
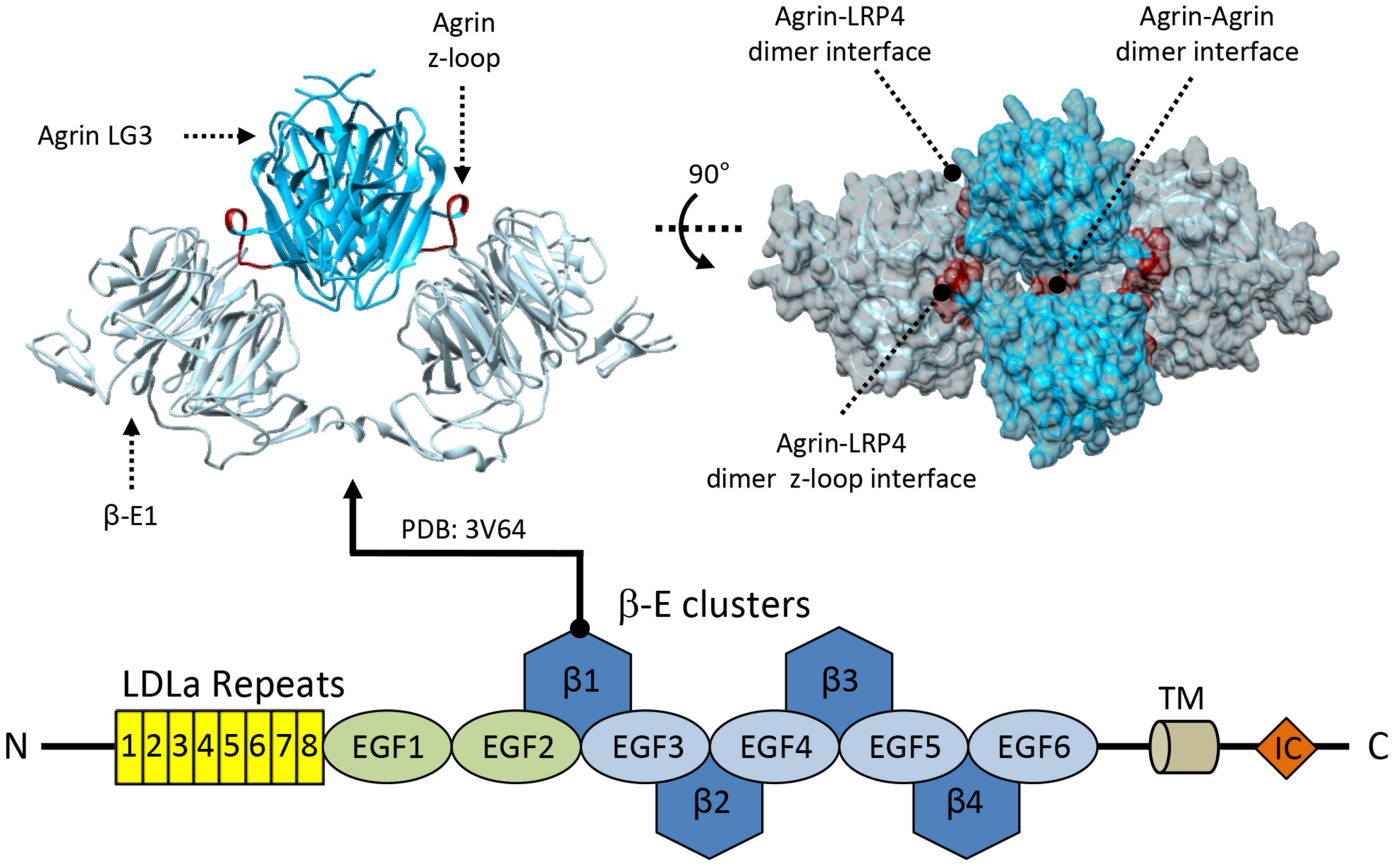
Structural information about LRP4. LRP4 interacts with agrin via its first β-propeller domain. The available crystal structure shows an overall tetrameric complex formed by each of the two molecules of agrin-LG3 (deep sky blue) simultaneously interacting with two LRP4 β-E1 monomers (light-gray) using two different binding sites. The primary agrin:LRP4 interface is mediated by the agrin z8-insert loop binding directly to the LRP4-β-E1 surface, shown in red. Additional secondary agrin:agrin and agrin:LRP4 contact points are highlighted in blue and yellow, respectively.

Conversely, the functionality of the cluster of four consecutive β-E domains is better understood, however clear roles have been proposed only for β-E1 and β-E3. In LRPs, β-E domains are the key sites for ligand recognition, a function which they seem to play also in the case of LRP4. Notably, agrin:LRP4 interactions heavily depend upon by the binding of the LRP4 β-E1 to a highly conserved, critical, NxI/V motif found on the agrin LG3 z8 loop (Zong et al., 2012). Crystal structures encompassing the first β-E1 in complex with the LG3 domain of agrin z8 (Zong et al., 2012; Figure 5) confirmed the importance of this motif located directly on the agrin z8 splice insert. This loop, which is untraceable in other structures (Stetefeld et al., 2004), is fully stabilized in the concave groove of the LRP4 β-E1 and constitutes the main contact site with the this domain of the co-receptor (Zong et al., 2012).

The agrin:LRP4 structures show a 2:2 hetero tetrameric arrangement (Figure 5), which was also confirmed by solution studies (Zhang et al., 2011; Zong et al., 2012). In the agrin:LRP4 complex, each agrin LG3z8 molecule contacts two LRP4 β-E1 using different surface areas. Very little agrin:agrin contacts were identified, and their functional significance was ruled out; no LRP4:LRP4 homodimeric contacts are present (Figure 5). This complex represents the essential recognition unit between the ligand and co-receptor proteins, although additional contact sites on LRP4 such as those involving the LDLa repeats were identified (Zhang et al., 2011).

Unlike what discussed previously, the functional role of the LRP4 β-E2 domain is less understood. Nonetheless, it likely plays an accessory function as spacer between the main agrin contact site (β-E1) and the region involved in the interaction with MuSK, localized in the β-E3 and β-E4 domains (Zhang et al., 2011). The β-E3 domain has been proposed to mediate direct LRP4:MuSK interactions. Accordingly, the deletion of this domain fully disrupts interactions between these two co-receptors, and abolishes MuSK phosphorylation induced by agrin stimulation (Zhang et al., 2011). On the other hand, the β-E4 domain seems to play a more complementary role as deletion experiments have shown that this domain minimally affects the LRP4 interactions with both MuSK and agrin *in vitro*. However, in absence of the LRP4 β-E4 domain the overall agrin-induced MuSK phosphorylation was found to be significantly reduced (Zhang et al., 2011). These findings indicate that while the LRP4 β-E4 may not be directly involved in binding, it may strongly influence the overall extracellular agrin:LRP4:MuSK conformational organization. As such, it could play a significant role in facilitating the correct positioning of the transmembrane and intracellular MuSK domains necessary for its activation.

## Modulation of Agrin Signaling by Proteolytic Cleavage

Also known as PRSS12 and motopsin, Neurotrypsin (NT) is a multi-domain extracellular serine-protease of the nervous system whose only know substrate is agrin (Gschwend et al., 1997; Yamamura et al., 1997). This protease plays a regulatory role at the NMJ by proteolytically processing agrin and, by doing so, modulating downstream signaling (Bolliger et al., 2010; Butikofer et al., 2011).

Two cleavage sites for NT, α, and β, have been located C-terminus to agrin's SEA and EGF4 domains, respectively (Reif et al., 2007; Figure 2A). Cleavage at the α site results in the generation of two fragments: a basal-lamina/membrane bound N-ter fragment carrying the vast majority of agrin's glycan moieties, and a ~110 kDa C-terminal soluble fragment. This last portion is further processed at the β site to generate 90 and 22 kDa fragments. While presented sequentially α to β, the order of cleavage in the native environment is currently unknown and could, instead, occur in parallel or in a β to α direction (Gisler et al., 2013). The exact function of the agrin cleavage products is uncertain, especially in regards to the N-terminal fragment. However, efficient agrin-induced AChR clustering seems to rely on the full 110 kDa C-terminal fragment encompassing both β-cleavage fragments (Bezakova and Ruegg, 2003; Bolliger et al., 2010). Interestingly the 22 kDa fragment, corresponding to the agrin LG3 domain, seems to be capable of stimulating neuronal branching in the CNS as well as inducing AChR clustering at the NMJ (with ~10^2^ to 10^3^-fold lower efficacy) (Bezakova and Ruegg, 2003; Matsumoto-Miyai et al., 2009).

## Neurotrypsin: Agrin's Regulatory Protease

Spanning 875 aminoacids, human NT is characterized by an N-terminal proline rich segment, a kringle domain (Kr), four scavenger receptor cysteine rich (SRCR) domains and a putative furin zymogen activation site preceding a C-terminal serine-protease domain (SP) (Proba et al., 1998; Figure 2D). Interestingly, the SRCR domains represent a point of divergence between the human and murine variants, as the first SRCR domain of the human protein seems to be absent in mice (Proba et al., 1998; Canciani et al., 2019).

Information on NT from a molecular standpoint mostly originates from three key papers published between 2007 and 2013 (Mitsui et al., 2007; Reif et al., 2008; Gisler et al., 2013). These form the basis of our biochemical understanding of NT, highlighting significant substrate specificity and optimal activity in pseudo-physiological conditions: pH~7–8 and 100–200 mM NaCl (Mitsui et al., 2007; Reif et al., 2008). Of particular interest is the reported modulation of NT activity by heparan sulfate (HS) glycosaminoglycans and by Ca^2+^ (Reif et al., 2008; Gisler et al., 2013). Given agrin's nature as a HS proteoglycan (Halfter, 1993; Burg et al., 1995; Winzen et al., 2003) alongside its dependency on Ca^2+^ for AChR clustering (Wallace, 1988), regulation of NT activity by these same factors offers an interesting point of convergence between agrin-mediated signaling and its proteolytic regulation. As such, it would be alluring to consider the possibility of HS and Ca^2+^ acting as coordinators to tie agrin cleavage to specific spatio-temporal constraints. This could not only determine selectivity and/or sequentiality of agrin α and β cleavage (Gisler et al., 2013), but also coordinate agrin processing at the NMJ to the formation of the full agrin:LRP4:MuSK complex.

**Figure 6:**
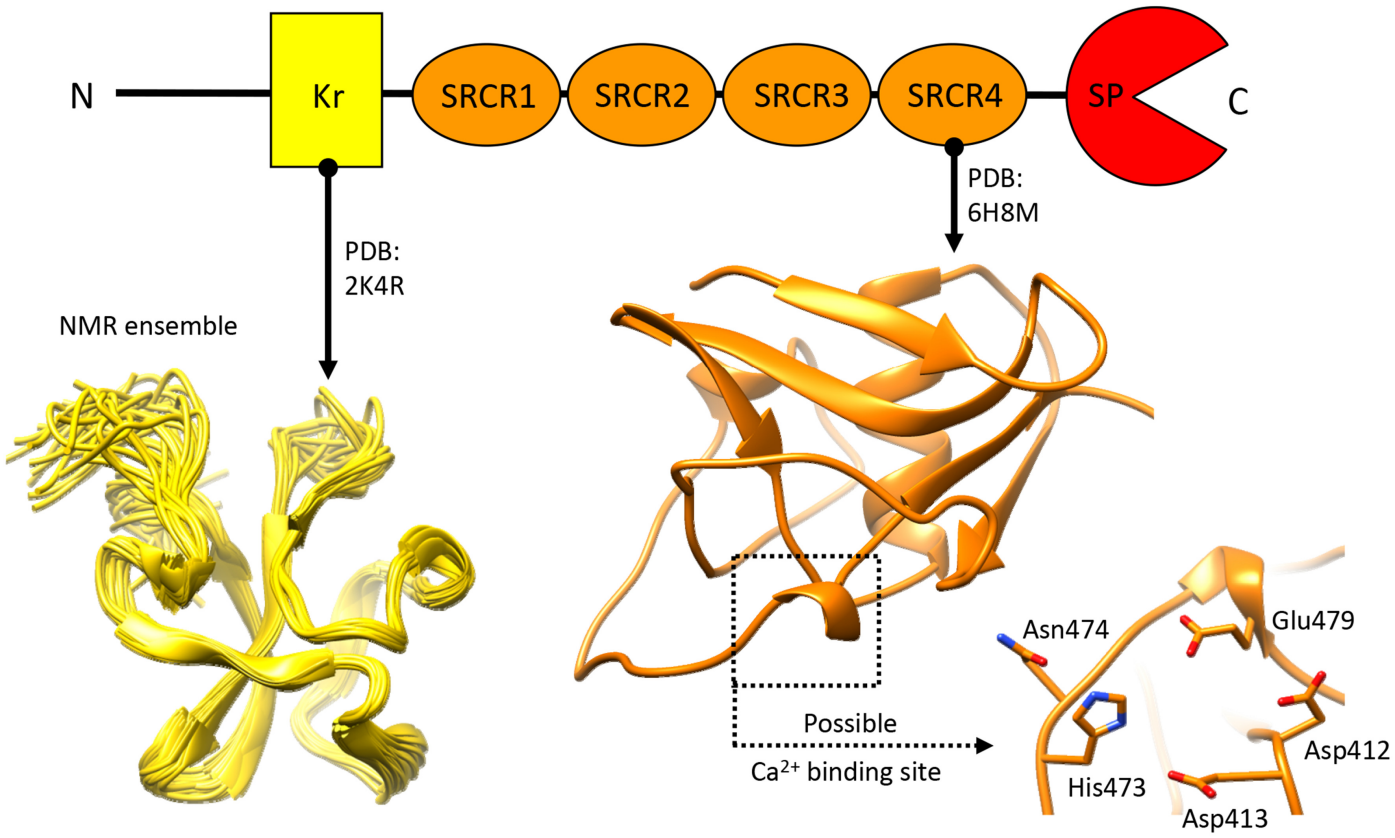
Structural information about Neurotrypsin. The NMR structure of the N-terminal Kr domain and the crystal structure of the Ca^2+^-binding rat SRCR3 domain (corresponding to human SRCR4 domain) are shown. At present, no other structural insights are available for this extracellular protease.

Knowledge pertaining to NTs structural organization is limited (Figure 6), as only the structures for the N-terminal Kr domain (Ozhogina et al., 2008) and the C-terminal SRCR domain (Canciani et al., 2019) proximate to the SP domain have been described. The former has been attributed with no specific function beyond a putative binding to the product of seizure related gene 6 (Mitsui et al., 2013). Similarly, precious little has been reported, so far, pertaining to the functional role of NT's SRCR domains. Realistically, these are likely to mediate protein-target interactions, and/or contribute to Ca^2+^ binding (Martinez et al., 2011; Canciani et al., 2019). Interestingly, in other proteins of the SRCR domain-bearing superfamily several domains of this type have been attributed with a Ca2^+^ dependant ligand recognition/binding function (Sarrias et al., 2005, 2007; Ojala et al., 2007; Reichhardt et al., 2019). While the limited molecular data on NT makes it currently difficult to determine whether this role is shared by NT's SRCR domains, the possibility is considerably intriguing.

Overall, adequate regulation of NT proteolytic modulation of agrin signaling seems to be essential to proper NMJ maintenance, as NT overexpression results in accelerated NMJ degeneration (Matsumoto-Miyai et al., 2009; Bolliger et al., 2010; Butikofer et al., 2011). Interestingly, this phenotype could be rescued by engineered cleavage-resistant fragments of agrin (LG2-LG3 C-terminal segment), hinting at the greater importance of the longer agrin fragments to NMJ formation and maintenance (Hettwer et al., 2014). This highlights the likely role of agrin's proteolytic processing in regulating the activation and duration of signaling from the agrin:LRP4:MuSK complex (Bolliger et al., 2010; Butikofer et al., 2011; Hettwer et al., 2014). Thus, regulation of agrin proteolysis offers an interesting therapeutic prospective for pathologies associated to neuromuscular degeneration. Modulation of NT activity, as well as agrin-derived therapeutics, could provide a means to delay or compensate the progression of the pathology at a NMJ level, and provide a retrograde positive signal counteracting, in part, the associated motor neuron loss (Hettwer et al., 2014; Boido et al., 2018; Li Z. et al., 2018).

## To Wnt or not to Wnt

Among the processes enabling formation of mature, functional, NMJs, the neural agrin-induced pathway for AChR clustering is perhaps the most investigated and best understood. It is not, however, the only means of inducing AChR clustering nor the only regulatory mechanism underlying NMJ formation (Li L. et al., 2018). Indeed, the processes governing the essential steps of AChR pre-patterning can, and do, operate in an agrin-independent capacity (Lin et al., 2001). As such, these likely rely on alternative mechanisms such as LRP4:MuSK “auto-activation” (Kim and Burden, 2008; Remedio et al., 2016) and Wnt mediated signaling (Zhang et al., 2012a; Messeant et al., 2017; Boëx et al., 2018).

The latter is particularly interesting as it evidences a link/coordination between AChR clustering, essential to NMJ formation, and the Wnt pathways. These regulate cellular differentiation and specialization via distinct signaling mechanisms: the “canonical” β-catenin stabilization regulating gene expression, and the “non-canonical” planar-cell-polarity (PCP) & Ca^2+^/Calmodulin pathways that influence cytoskeletal rearrangement (Komiya and Habas, 2008; Nusse and Clevers, 2017; Ng et al., 2019). Both involve binding of Wnt molecules to transmembrane Frizzled receptors but “canonical” Wnt signaling requires additional interaction with co-receptors of the low density lipoprotein class LRP5/6 (Komiya and Habas, 2008; Macdonald and He, 2012; Nusse and Clevers, 2017). Interestingly, at the NMJ these pathways seem to exist in a sort of canonical/non-canonical dynamic equilibrium, as canonical Wnt signaling seems to be required for AChR pre-patterning/aneural clustering (Messeant et al., 2017) but negatively regulates Rapsyn-induced AChR clustering (Wang J. et al., 2008). Conversely, favoring of Wnt non-canonical signaling via Dickkopf 1 (Dkk1) results in impaired pre-patterning alongside aberrant motor neuron branching and NMJ formation (Messeant et al., 2017). Finally β-catenin gain-of-function or loss-of-function mice models display similarly perturbed NMJ formation (Wu et al., 2010; Liu et al., 2012). Collectively, these observations highlight how the Wnt molecules and their signaling pathways are important for NMJ formation.

However, their mode of signaling at the NMJ is uncertain and remains matter of debate (Messeant et al., 2015; Remedio et al., 2016; Boëx et al., 2018). Reasonable hypotheses include signaling via canonical Frizzled:LRP5/6, mediation by LRP4 (necessary for prepatterning) via an unidentified receptor, or (more plausibly) mediation via the LRP4:MuSK pathway. This last theory bears significant merit as both LRP4 and MuSK are necessary (in mice) for NMJ prepatterning/formation and several Wnt molecules were seen to directly influence AChR clustering (Henriquez et al., 2008; Zhang et al., 2012a; Messeant et al., 2015). Unsurprisingly, a subset of Wnt molecules were found to bind directly to MuSK (Wnt4, Wnt11, Wnt9a) and LRP4 (Wnt9a, Wnt11) (Strochlic et al., 2012; Zhang et al., 2012a). Furthermore, MuSK is known to interact intracellularly with the Wnt mediator Disheveled (Dsh), that sits at the crossroads between Wnt canonical and non-canonical pathways (Luo et al., 2002; Komiya and Habas, 2008). Finally, Wnt canonical pathway inhibitors such as Dkk1, sclerostin (SOST) and bone morphogenic proteins (BMPs) were reported to bind to LRP4, thus providing an additional, possible, layer of modulation (Shen et al., 2015). As such, the cross-talk between AChR clustering and Wnts is likely coordinated and mediated through the LRP4:MuSK complex.

## The “Structural” Possibility of Wnt-LRP4:MuSK Cross-Talk

**Figure 7:**
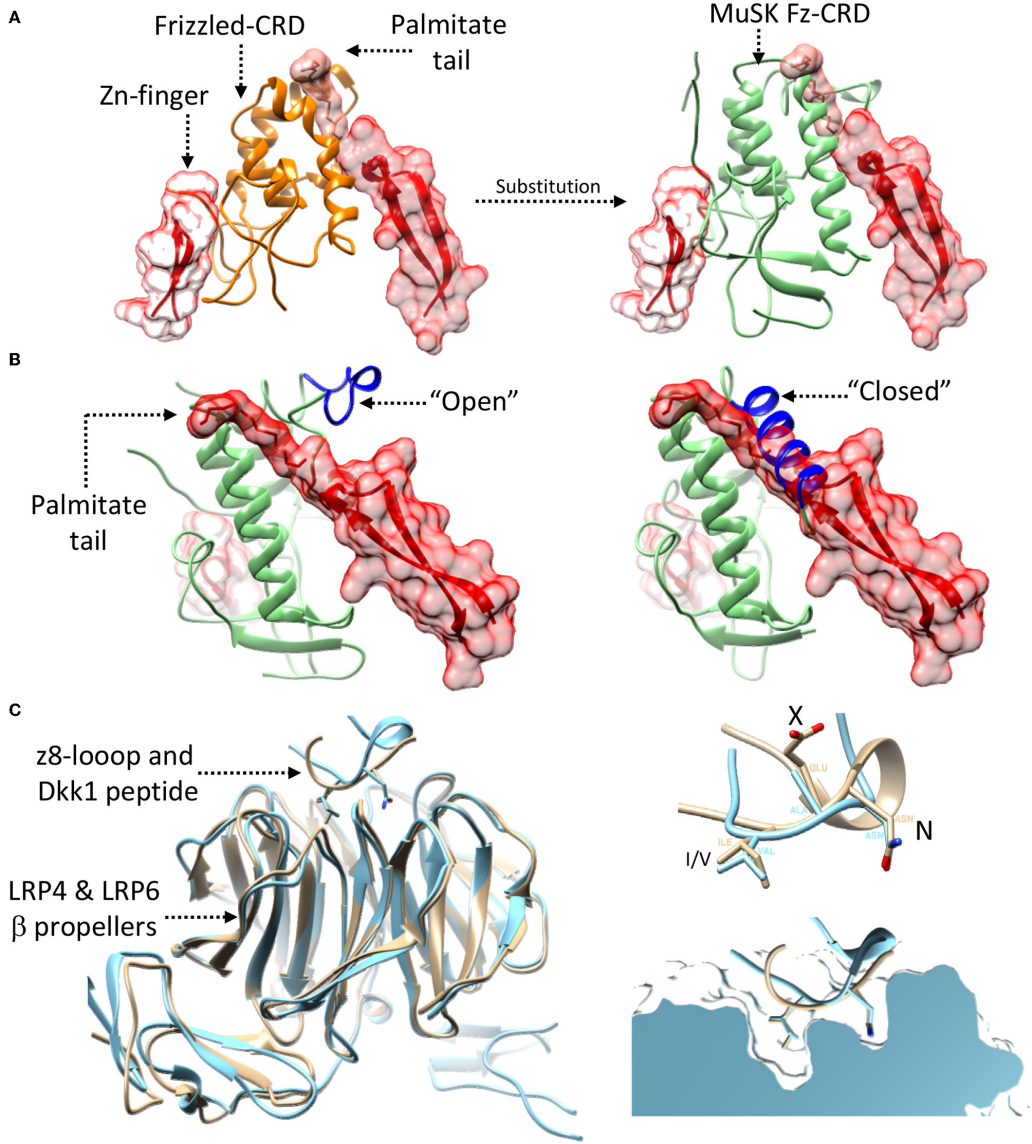
Possibility of Wnt-agrin:LRP4:MuSK cross-talk. **(A)** The interaction (left-panel) between Wnt's (red) and Frizzled domains (orange) relies on two opposed interfaces that accommodate contemporaneously a palmitate tail and a Zn-finger domain. Substituting (right-panel) the Frizzled domain with the MuSK Fz-CRD (light-green) shows how this domain of MuSK could accommodate the same type of opposed binding interfaces. **(B)** The crystal structure of the MuSK Fz-CRD (also shown in Figure 4) presents two alternative conformations; an “open” conformation (left), possibly capable of accommodating the Wnt palmitate tail, and a “closed” one, likely not conductive to Wnt binding (right). “open” and “closed” states are determined the by the stabilization of an α-helix from an otherwise disordered loop (shown in blue). **(C)** A superposition (left) of the agrin:LRP4 z8 loop complex (cyan) with an LRP6:Dkk1 N-terminal peptide complex (beige) shows the structural similarity of the interaction, hinting the possibility of cross-talk. Both the agrin z8-loop and the Dkk1 peptide interact with their target via a conserved NxI/V motif (top-right). A cross section view into the binding pocket of LRP4 (top) for the agrin-z8 loop (cyan) shows how it could also accommodate the Dkk1 peptide (beige) and allow for cross-talk between the two signaling pathways.

While the exact details on how and where Wnt-LRP4:MuSK cross-talk occurs remain uncertain, the structural and molecular data available on MuSK, LRP4, and agrin can provide possible hints. In this context the extracellular Fz-CRD of MuSK represents the most obvious link, in merit of its homology to extracellular Frizzled domains known mediate Wnt binding (Masiakowski and Yancopoulos, 1998; Stiegler et al., 2009). Deletion experiments have tentatively established a role for this domain in mediating Wnt signaling. However, while in Zebrafish models its importance is more evident (Jing et al., 2009) the corresponding mouse models have provided eloquent but less obvious results (Messeant et al., 2015; Remedio et al., 2016). Nonetheless, a structural comparison of the rat MuSK Fz-CRD (Stiegler et al., 2009) with the mouse Wnt3-Frizzled8 complex (Hirai et al., 2019) seems to define a clearer picture. This shows how the MuSK Fz-CRD might be conductive to Wnt binding by accommodating the diametrically opposed binding of the Wnt palmitate tail and Zn-finger domain (Figure 7A) much like the already available structures of Wnt:Frizzled complexes (Janda et al., 2012; Hirai et al., 2019). Interestingly, the MuSK Fz-CRD adopts two distinct conformations in crystal structures (Stiegler et al., 2009), respectively likely or unlikely to bind Wnts (Figure 7B). These could represent a “structural switch” between Wnt- and agrin-mediated LRP4:MuSK activation and thus correlate to a shift from pre-patterning to NMJ maturation and stabilization.

Further insight into Wnt-NMJ cross-talk can be gleaned from the interaction between neural agrin and LRP4. The available structure highlights how the agrin z8 splice insert binds to the β-E1 domain of LRP4 via the conserved NxI/V motif (Zong et al., 2012). This mirrors the interactions observed between Wnt mediators LRP5/6 and canonical Wnt pathway inhibitors such as Dkk1 (Choi et al., 2009; Bourhis et al., 2011). Additional evidence can be found in a structure of the β-E1 domain of LRP6 in complex with an N-terminal peptide of Dkk1 (Cheng et al., 2011). A comparison with the agrin:LRP4 structure highlights how the Dkk1 peptide and the agrin z8-loop interact with LRP4/5/6 β-E domains in a distinctly homologous fashion (Figure 7C), possibly opening the discussion to further speculation. Given the interaction similarities with LRP4/5/6 between agrin and Wnt-canonical pathway inhibitors, it could be possible that agrin itself might act in a similar capacity, either through LRP4 or via cross-interaction on LRP5/6. In such a way, neural agrin, present only at motor neuron-muscle fiber contact sites, could act as a switch signal to shift from pre-patterning to NMJ maturation. In this context z8-bearing agrin would act to both to stimulate AChR clustering via MuSK and induce a localized shunt to non-canonical Wnt signaling via an LRP co-receptor. This canonical/non-canonical Wnt modulation could be necessary to shepherd the restricted post-synaptic cytoskeletal remodulations associated to NMJ maturation.

A key difference between LRP4 and LRP5/6 lies in the quaternary arrangement of the ligand:co-receptor structures. The agrin:LRP4 structures show a 2:2 hetero tetrameric arrangement (Figure 5; Zhang et al., 2011; Zong et al., 2012) that was never observed in homologous LRP5/6. Such organization may underline the specific function of the agrin:LRP4 interaction at NMJs, further highlighting the simultaneous promiscuity of this scavenger co-receptor molecule and its context-specific functions required during NMJ development.

Elaborate theories notwithstanding, the contribution of Wnt signaling to NMJ formation/maturation seems significant, especially at the prepatterning stage. Furthermore, the likely coordination with agrin:LRP4:MuSK-induced AChR clustering evidences the necessarily interconnected signaling/regulatory network underlying the proper formation of NMJs.

## Is an “Even Bigger Picture” Missing?

A significant number of additional “co-factors” contribute to the regulation of agrin:LRP4:MuSK signaling in NMJ formation. For example, both Ca^2+^ and heparin play significant roles in the regulation of NT-mediated agrin cleavage, thus modulating agrin-induced AChR clustering (Reif et al., 2008; Bolliger et al., 2010; Gisler et al., 2013). Additional regulatory contributions by these two co-factors extends beyond their influence on NT activity. Notably, the influence of heparin or heparan sulfates could mediate regulation of Wnt-mediated events, as Wnts were reported to bind heparin/heparan sulfates alongside other extracellular matrix components (Reichsman et al., 1996; Berendsen et al., 2011; Tran et al., 2012; Gao et al., 2016). Furthermore, in the context of muscle tissue regeneration, Wnt pathways were seen to undergo some degree of regulation on the basis of the degree of sulfonation of heparan sulfates (Tran et al., 2012). On the other hand, Ca^2+^ was seen to be necessary for agrin-induced AChR clustering, likely owing to stabilization of agrin LG3-z8:LRP4 interactions (Stetefeld et al., 2004; Zong et al., 2012). Furthermore, downstream signaling from MuSK also seems to require intracellular Ca^2+^ currents (Borges et al., 2002). These could be the consequence of a localized, ACh induced, extracellular Ca^2+^ influx, and/or the result of the internalization of LRP4:MuSK, as LDLa domains are known to coordinate Ca^2+^ ions (Brown et al., 1997; Fass et al., 1997; Borges et al., 2002). As such, alterations in LRP4:MuSK internalization in presence of ligands such as agrin (Zhu et al., 2008) could serve in a modulatory capacity to regulate Ca^2+^ currents and coordinate extracellular-intracellular signaling.

The complete regulatory picture is, however, significantly more intricate seeing contributions from several additional components. These accessory molecules include such as Perlecan, α-dystroglycan, laminins, and even the amyloid precursor protein (APP) (Choi et al., 2013; Stanga et al., 2016; Li L. et al., 2018). This last one is particularly interesting as its contributions to agrin:LRP4:MuSK activation and presynaptic specialization (Choi et al., 2013) are emblematic of the elaborate nature of the signaling network surrounding NMJ formation.

## Disease-Related Variants in NMJ Signaling Proteins and Their Implications

Specific and fundamental roles are played by each NMJ-associated protein in the differentiation and specialization of both pre- and post-synaptic terminals. Unsurprisingly mutations in genes encoding for those proteins can result in the impairment of the previously described pathways. This can, in turn, give rise to several phenotypically different muscle-weakening diseases. Congenital myasthenic syndromes (CMS) are one such group of muscle diseases. They are generally associated to dominant or recessive autosomal Mendelian mutations found in ~32 genes encoding for both pre- and post-synaptic NMJ proteins (Finsterer, 2019). The overall prevalence of CMS is about 1/10 that of the acquired autoimmune form of an analogous pathology called *myasthenia gravis* (MG, described prevalence 25–125/1.000.000) (Gilhus et al., 2019). Diagnosis of myasthenic syndromes is particularly challenging as the predominant, clinically observable characteristic is muscle weakness, a phenotype commonly associated to a wide variety of other muscle-associated diseases.

## CMS Mutations Can Affect the Agrin:LRP4:MuSK Triad

Most CMS associated mutations can be found on genes encoding for AChRs, which yield abnormal products as a consequence of non-sense or frame-shift alterations. However, in the last few years, several mutations were identified that directly affect the agrin:LRP4:MuSK pathway (Table 1). Among these, missense CMS mutations in the third β-propeller of LRP4 (p.Glu1233Ala; p.Arg1277His) directly compromise interactions with MuSK (Ohkawara et al., 2014; Selcen et al., 2015). Unfortunately, as the structure of this domain of LRP4 is not available, the exact impact and structural ramifications of these mutations on LRP4:MuSK interactions remain unknown.

**Table 1:** List of pathogenic CMS mutations found in the NMJ signaling proteins described in this review.

**Protein**	**Mutation**	**Location of mutation**	**References**
MuSK	p.Asp38Glu	Ig1 domain	Gallenmuller et al., 2014
	c.220insC	Ig1 domain	Chevessier et al., 2004
	p.Asn103Ser	Ig1 domain	Giarrana et al., 2015
	c.467delA	Ig1 domain	Maggi et al., 2013
	p.Arg166^*^	Ig2 domain	Giarrana et al., 2015
	p.Pro344Arg	Fz-CRD	Mihaylova et al., 2009
	p.Met605Ile	TK domain	Maselli et al., 2010
	p.Pro650Thr	TK domain	Luan et al., 2016
	p.Ala727Val	TK domain	Maselli et al., 2010
	p.Val790Met	TK domain	Chevessier et al., 2004
	p.Ile795Ser	TK domain	Luan et al., 2016
	p.Met835Val	TK domain	Ben Ammar et al., 2013
LRP4	p.Glu1233Ala	β-E3 domain	Selcen et al., 2015
	p.Arg1277His	β-E3 domain	Ohkawara et al., 2014
Agrin	p.Gly76Ser	NtA domain	Nicole et al., 2014
	p.Asn105Ile	NtA domain	Nicole et al., 2014
	p.Gln353^*^	follistatin-like 3 domain	Maselli et al., 2012
	p.Gly1709Arg	LG2 domain	Huze et al., 2009
	p.Val1727Phe	LG2 domain	Maselli et al., 2012
	p.Ala1768Pro	LG2 domain	Karakaya et al., 2017
	p.Gly1875Arg	LG3 domain	Nicole et al., 2014
Dok7	p.Ser45Leu	PH domain	Ben Ammar et al., 2010
	p.Val116Met	PH domain	Ben Ammar et al., 2010
	p.Pro146Leu	PTB domain	Ben Ammar et al., 2010
	p.Leu157Arg	PTB domain	Ben Ammar et al., 2010
	p.Gly171Arg	PTB domain	Ben Ammar et al., 2010
	p.Gly172Arg	PTB domain	Ben Ammar et al., 2010
	p.Gly180Val	PTB domain	Ben Ammar et al., 2010
	p.Arg201^*^	PTB domain	Beeson et al., 2006
	c.1124TGCC-DUP		Beeson et al., 2006
Rapsyn	p.Leu14Pro	TPR1 domain	Ohno et al., 2002
	p.Val45Leu	TPR2 domain	Maselli et al., 2007
	p.Asn88Lys	TPR3 domain	Ohno et al., 2002
	p.Glu162Lys	TPR5 domain	Maselli et al., 2007
	p.Arg164Cys	TPR5 domain	Muller et al., 2006
	p.Tyr269^*^	TPR7 domain	Dunne and Maselli, 2003
	p.Leu283Pro	linker between TPR7 and coiled coil	Muller et al., 2006

For each mutation, the protein domain that contains the mutated residue is reported. Stars (^*^) indicate premature polypeptide termination due to insertion of stop codons in the mutated gene.

Upstream of LRP4 the pathway can be affected by means of mutations in the N- and C-terminal regions of agrin. Missense mutations in the agrin NtA domain (p.Gly76Ser, p.Asn105Ile) (Figure 3) result in a reduction of agrin-induced AChR clustering *in vitro* (Nicole et al., 2014), likely as a result of altering interactions with laminin, which anchors agrin to extracellular matrix (ECM).

On the other hand, mutations in C-terminal LG2 and LG3 domains are likely to hamper binding to LRP4, and result in NMJ formation defects caused by reduced LRP4:MuSK activation. Recently, the pathogenic agrin missense mutations p.Gly1709Arg (Huze et al., 2009) and p.Val1727Phe (Maselli et al., 2012) were mapped on the structure of its LG2 domain (Figure 3). These were seen to localize, respectively, in proximity to the y splicing insertion site and on the inner face of a β-strand contributing to this domain's jelly-roll fold (Rudell et al., 2019). While direct interactions between agrin LG2 domain and LRP4:MuSK have not been reported, the importance of this domain in agrin-mediated signaling at the NMJ is evident. Notably, its absence results in a several-fold reduction of agrin efficacy in inducing AChR clustering (Bezakova and Ruegg, 2003). Such contributions could be attributed to the roles proposed for this domain in mediating the binding of agrin to proteoglycans (Gesemann et al., 1996, 1998; Bezakova and Ruegg, 2003), as their presence on the muscle membrane is known to be essential to agrin function at the NMJ (Ferns et al., 1993). Accordingly, both LG2 localized mutations affect agrin glycan-binding properties, likely leading to the correlated CMS phenotypes (Rudell et al., 2019). Based on *in silico* modeling it has been proposed that this might depend on subtle structural alterations affecting the positioning and functionality of the flexible loop bearing the y4 splice insert (Rudell et al., 2019). It is likely, however, that the contributions of the agrin LG2 domain to LRP4:MuSK activation depend just as much on the overall domain architecture. This could account for the more severe traits correlated to the p.Val1727Phe substitution, especially given that of the two mutations only this one results in hampering of AChR clustering (Huze et al., 2009; Maselli et al., 2012; Rudell et al., 2019). However, the question as to whether the LG2 domain exclusively mediates binding to proteoglycans or might also be responsible for direct LRP4:MuSK interactions has yet to be addressed. Nonetheless, the LRP4-α-dystroglycan mediated effect of non-neural (z-) agrin on chondrocytes, seems to suggest a more complex and nuanced role. One where glycan-binding and agrin:LRP4 interactions seem to converge (Eldridge et al., 2016).

Missense mutations affecting MuSK mainly result in receptor inactivation and are predominantly localized on the intracellular kinase domain (Chevessier et al., 2004; Ben Ammar et al., 2010; Maselli et al., 2010; Luan et al., 2016). However, a small number of missense mutations were found to affect the MuSK ectodomain; two in the Ig1 domain (p.Asp38Glu; p.Asn103Ser) (Gallenmuller et al., 2014; Giarrana et al., 2015) and one in the Fz-CRD (p.Pro344Arg) (Mihaylova et al., 2009). The Ig1 variant p.Asp38Glu is a surprisingly conservative mutation localized on the external surface of the domain. However, this pathogenic mutation likely interferes with MuSK activation indirectly, as its structural proximity to the aminoacid Ile96 (mentioned previously) is likely to disrupt the essential role of that residue in LRP4:MuSK co-receptor interactions (Figure 4) (Gallenmuller et al., 2014). On the other hand, the p.Pro344Arg mutation on Fz-CRD is significantly less conservative, and likely results in domain misfolding collectively destabilizing the MuSK receptor (Milhem et al., 2015). In both cases, these mutations further emphasize the importance of these two extracellular domains in MuSK activation and signaling. Unfortunately, the limited availability of detailed molecular insights regarding MuSK architecture and activation currently hampers a comprehensive evaluation of the impact of disease-causing mutations at the protein level. This makes all but impossible the task of functionally correlating protein alterations with corresponding clinical symptoms. Future work in this direction has therefore the potential to improve diagnostics, facilitate the evaluation of prognosis for each specific case, and also allow the development of innovative, dedicated therapeutic strategies.

## The Role of the Auto-Antibodies Involved in Myasthenia Gravis

A vast number of myasthenic syndromes are caused by impaired AChR and/or agrin:LRP4:MuSK signaling as a consequence of genetic mutations. This is, however, is not the case for MG, the most common myasthenic syndrome. This rare autoimmune disease is caused by the presence of autoantibodies that target specific epitopes on NMJ proteins, thus impairing their physiological functions in neuromuscular pathways (Koneczny et al., 2013; Gilhus et al., 2019). These autoantibodies lead to NMJ disassembly causing severe impairment in muscle contraction (Carr et al., 2010), with symptom severity dependant on the type of muscle involved as well as the age of onset. Of all the autoantibodies involved in MG the most widespread, and best characterized, are the class 1–3 IgG anti-AChR autoantibodies (Lefvert et al., 1981; Rodgaard et al., 1987; Slater, 2008; Gilhus et al., 2019; Koneczny and Herbst, 2019). These are known to reduce synaptic function through several mechanisms, such as: direct inhibition of AChR mediated ACh signaling (Rodgaard et al., 1987; Gomez et al., 2010), increased receptor endocytosis (Bursztajn et al., 1983; Clementi and Sher, 1985), and activation of complement immune responses (Howard, 2018). While anti-AChR autoantibodies may be the most common, MG can be the result of different autoantibodies that target the agrin:LRP4:MuSK pathway.

One such example is a set of class 4 Anti-MuSK IgG autoantibodies, found to target the extracellular region of MuSK (Niks et al., 2007; Koneczny et al., 2013). Interestingly, unlike other IgG classes, IgG4 antibodies do not activate the complement immune response but, in physiological conditions, can display an anti-inflammatory effect (Trampert et al., 2018). However, in pathological conditions, such as in MG, they are known to inhibit protein:protein interactions impairing downstream signaling pathways. Accordingly, epitope mapping on MuSK showed that anti-MuSK autoantibodies target predominantly domains involved in LRP4:MuSK interactions: the Ig1 and Fz-CRD (Huijbers et al., 2016; Figure 2B). Anti-Ig1 autoantibodies were initially found to negatively affect MuSK phosphorylation (Koneczny et al., 2013; Huijbers et al., 2016), likely through direct impairment of LRP4:MuSK interactions. However, given the limited availability of *in vitro* studies on these autoantibodies, they could just as well interfere with MuSK Ig1:Ig1 homodimerization. Interestingly, very recent studies have also shown that anti-MuSK autoantibodies may present different valences and pathological outcomes (Huijbers et al., 2019). Bivalent anti-Ig1 can induce AChR clustering, possibly triggering receptor conformational changes allowing the productive interaction of the two Ig1 domains without intervention by agrin nor LRP4. Conversely, monovalent anti-Ig1 were found to block the LRP4:MuSK interaction and consequently AChR clustering and differentiation of the presynaptic terminal. Finally, Anti Fz-CRD antibodies were also observed in patients (Takamori et al., 2013), and their presence may indirectly explain the importance of the Fz-CRD in Wnt-mediated MuSK activation.

In addition to anti-MuSK autoantibodies, both anti-agrin and anti-LRP4 autoantibodies have also been found in MG patients (Higuchi et al., 2011; Pevzner et al., 2012; Zhang et al., 2012b), but the precise mechanisms underlying their pathogeneicity are still unknown. Recent studies have shown that treatment with serum from MG patients can reduce canonical AChR clustering in C2C12 myotubes. Furthermore, this serum was also capable of inhibiting agrin:LRP4 interactions *in vitro* (Higuchi et al., 2011; Pevzner et al., 2012; Zhang et al., 2012b, 2014). Unfortunately, epitope mapping has not been reported yet for these autoantibodies. Nonetheless, based on the available molecular knowledge, an intuitive guess could be that they might target an epitope in close proximity to the agrin z8 loop or even the β-E1 domain of LRP4.

## Harnessing NMJ Molecules for Next-Generation Therapeutics

Current therapies for myasthenic diseases are based on two strategies: modulation of the immune system via immunosuppressant therapies and plasmapheresis, or a more direct intervention on the processes governing NMJ formation and stabilization (Koneczny and Herbst, 2019). Here, we will focus on the strategies revolving around NMJ targeted therapy.

Several therapeutic approaches have been studied to attempt stabilization and regulation of functionally compromised AChRs. In autoimmune MG caused by anti-AChR autoantibodies, the predominant strategy is based on acetylcholinesterase (AChE) inhibitors. These small-molecules reduce ACh metabolism increasing neurotransmitter retention in the synaptic cleft. This has the benefit of compensating for the reduced number of AChR clusters in stable NMJs (Sanders et al., 2016). However, in patients with anti-MuSK antibodies, the use of AChE inhibitors was seen to have a deleterious effect, worsening the severity of myasthenic symptoms (Hatanaka et al., 2005; Evoli et al., 2008). Given that this form of MG is caused by a reduction in MuSK-induced AChR clustering, this unexpected effect could be a consequence of excessive AChR cluster dispersion induced by the prolonged permanence of ACh at the NMJ.

In light of these observations, alternative therapeutic approaches are a necessity. One possibility takes into consideration the use of recombinant agrin constructs incorporating the C-terminal region of neural (z+) agrin known to induce AChR clustering (Hettwer et al., 2014; Li Z. et al., 2018). Specifically, a soluble C-terminal agrin fragment (NT1654) (Hettwer et al., 2014) spanning the LG2-LG3 (z8) domains. This recombinant protein, engineered to resist NT cleavage, was seen to hold significant potential in stabilizing NMJ's and alleviating autoimmune MG (Li Z. et al., 2018). Interestingly, agrin's possible therapeutic applications seem to extend beyond myasthenic syndromes to other pathologies associated with muscle degeneration. Notably, the previously mentioned construct was seen to positively affect models of spinal muscular atrophy (SMA) (Boido et al., 2018) and sarcopoenia (Hettwer et al., 2014). Similarly, a muscle-agrin (y0z0) derived construct, termed mini-agrin (Moll et al., 2001), is worthy of mention for its potential in alleviating certain types muscular dystrophies (Moll et al., 2001; Bentzinger et al., 2005; Qiao et al., 2005, 2018; Meinen et al., 2011).

In parallel to these burgeoning recombinant agrin possibilities, new immunotherapeutic strategies have been proposed to protect post-synaptic nerve termini through stabilization of active MuSK conformations. These methods hold a great potential for possible therapeutic intervention beyond CMS and MG, extending to other NMJ degenerative scenarios such as amyotrophic lateral sclerosis (ALS) or spinal muscular atrophy (SMA). Indeed, recent studies performed on ALS transgenic mice revealed that the degenerative process typical of the disease starts with the destruction of the NMJ and progresses along the axon, ultimately damaging the motor neuronal soma in a “dying back manner” (Dupuis and Loeffler, 2009; Tremblay et al., 2017; Martineau et al., 2018). This amount of evidence suggests that intervening on the factors deputed to preserve NMJ integrity and function may represent a promising strategy, and initial attempts yielded encouraging results (Krakora et al., 2012; Perry et al., 2017). Very recent results from ALS animal models have shown that treatment with a bivalent anti-Fz-CRD antibody seems to delay disease progression, showing reduction of muscle denervation and increased lifespan (Cantor et al., 2018). While the therapeutic outcome of currently implemented strategies is affected by limitations (Sengupta-Ghosh et al., 2019), these results encourage research efforts toward the possibility of using exogenous MuSK agonists to restore NMJ functionality (Glass, 2018).

## Conclusions

**Figure 8:**
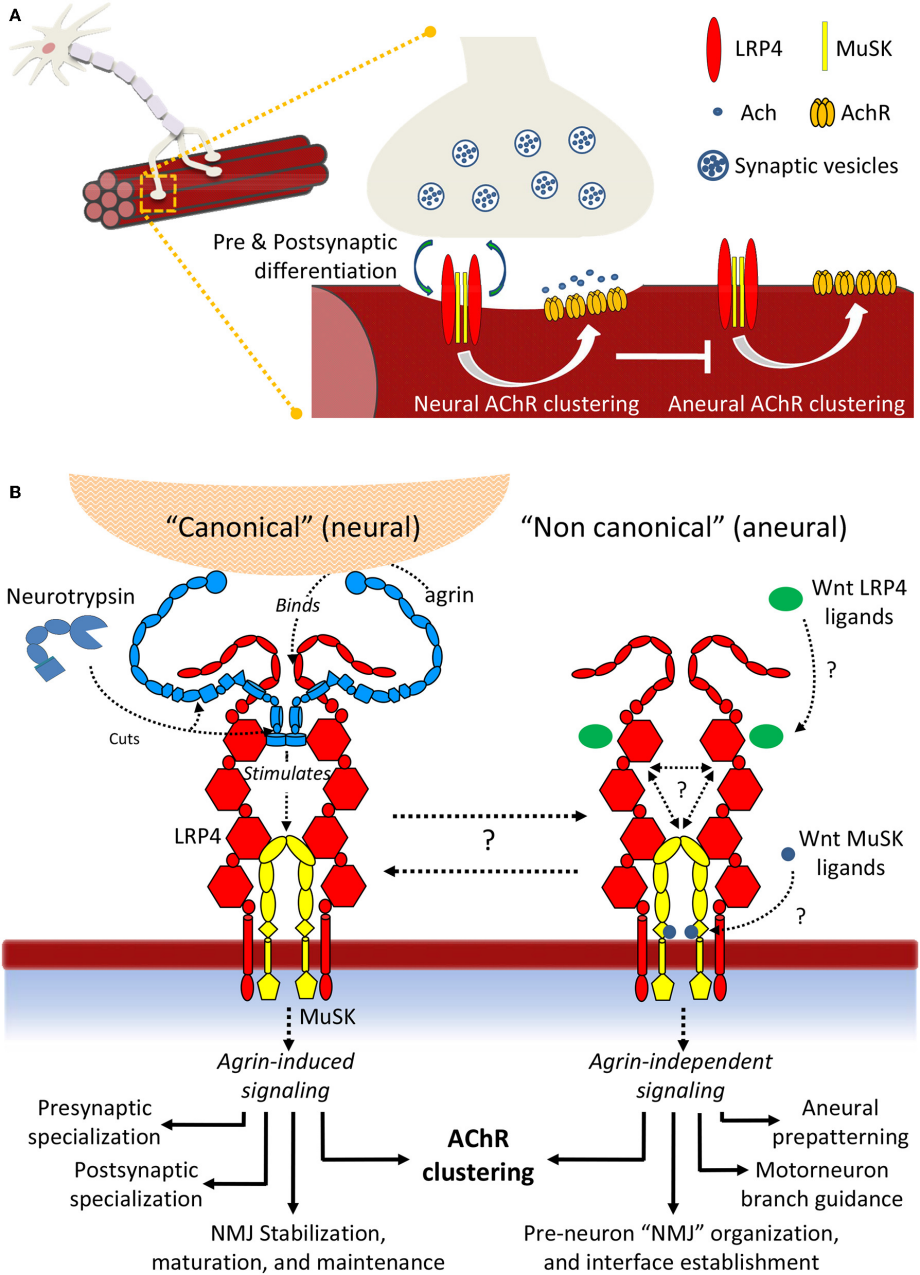
Summary scheme of LRP4:MuSK signaling. **(A)** Signaling events differentially induce AChR clustering toward either NMJ formation and motor neuron innervation or toward formation of aneural clusters. **(B)** Details of neural and aneural NMJ signaling via LRP4:MuSK activation. In canonical (agrin-dependent) NMJ signaling, binding of agrin to co-receptor LRP4 stimulates activation of MuSK and downstream effector functions (left). In this pathway, Neurotrypsin likely operates as a regulator through specific agrin cleavage at α and β sites; non-canonical (i.e., agrin-independent) events such as cross-talk with Wnt and BMP signaling pathways also trigger MuSK activation, possibly through direct binding of Wnt ligands and inhibitors to MuSK Fz-CRD or to LRP4 β-E domain clusters. The downstream effector functions differ into a spectrum that highlights numerous aneural regulatory events that could be particularly critical prior to motor neuron innervation and NMJ formation.

The last decade has witnessed incredible advances in our molecular knowledge surrounding NMJ formation and signaling, both in homeostasis and disease. Taken together, the accumulated insights depicts a non-linear scenario, characterized by unprecedented molecular complexity and a variety of poorly characterized molecular interactions. This results in a large number of yet unresolved questions (Figure 8). Regarding “canonical” agrin-dependent signaling, a key question resides in the molecular mechanisms triggering MuSK conformational changes: formation of the agrin:LRP4 complex clearly impacts the surrounding receptor environment, resulting in MuSK autophosphorylation. But what are the actual driving forces behind such activation? What are the contributions of molecular crowding, oligomerization, conformational stability, and flexibility to this fundamental event? A second, essential aspect underlines the precise functions of Neurotrypsin and its possible regulatory roles. The intriguing exclusive specificity for agrin, juxtaposed to the ability to process two different aminoacid sequences may suggest distinct *in vivo* regulatory functions, supporting the observed different impacts of NT at the NMJ and in the CNS (Molinari et al., 2002; Wang Y. et al., 2008; Butikofer et al., 2011).

Even wider is the landscape of open questions underlying agrin-independent (i.e., non-canonical) MuSK signaling at the NMJ. The cross-talks with other developmental pathways such as Wnt and BMP are supported by numerous insights at the cellular level. However, a molecular validation of how specific ligands may stimulate or abolish MuSK signaling through direct interactions with this TK receptor and/or its co-receptor LRP4 is still missing. Likewise, we are still far from being able to describe the biological “big picture” of how NMJs are formed, stabilized and functioning at the molecular level. Quantitative biochemical and biophysical data accounting for precise protein:protein interactions integrated with molecular structure data on large, flexible molecular assemblies [also possibly taking advance of the most recent *in vitro* and *in situ* methods of structural investigation (Palamini et al., 2016; Oikonomou and Jensen, 2017)] will be key to advancing to the next level in our comprehension of this essential, and still very enigmatic, synaptic connection.

## Author Contributions

All authors listed have made a substantial, direct and intellectual contribution to the work, and approved it for publication.

### Conflict of Interest

The authors declare that the research was conducted in the absence of any commercial or financial relationships that could be construed as a potential conflict of interest. The handling Editor declared a past co-authorship with one of the authors FF.
